# Polydiacetylene (PDA) Embedded Polymer-Based Network Structure for Biosensor Applications

**DOI:** 10.3390/gels11010066

**Published:** 2025-01-15

**Authors:** Huisoo Jang, Junhyeon Jeon, Mingyeong Shin, Geonha Kang, Hyunil Ryu, Sun Min Kim, Tae-Joon Jeon

**Affiliations:** 1 Industrial Science and Technology Research Institute, Inha University, 100 Inha-ro, Michuhol-gu, Incheon 22212, Republic of Korea; hsjang@inha.ac.kr; 2 Biohybrid Systems Research Center (BSRC), Inha University, 100 Inha-ro, Michuhol-gu, Incheon 22212, Republic of Korea; junh.jeon@inha.edu (J.J.); hyunil.ryu@inha.ac.kr (H.R.); 3 Department of Mechanical Engineering, Inha University, 100 Inha-ro, Michuhol-gu, Incheon 22212, Republic of Korea; elldica@inha.edu; 4 Department of Food and Nutrition, Inha University, 100 Inha-ro, Michuhol-gu, Incheon 22212, Republic of Korea; sin020719@inha.edu; 5 Department of Biological Sciences and Bioengineering, Inha University, 100 Inha-ro, Michuhol-gu, Incheon 22212, Republic of Korea; 6 Department of Biological Engineering, Inha University, 100 Inha-ro, Michuhol-gu, Incheon 22212, Republic of Korea

**Keywords:** colorimetric biosensors, polydiacetylene, hydrogel, polymeric structure, synthetic polymers, point-of-care detection

## Abstract

Biosensors, which combine physical transducers with biorecognition elements, have seen significant advancement due to the heightened interest in rapid diagnostic technologies across a number of fields, including medical diagnostics, environmental monitoring, and food safety. In particular, polydiacetylene (PDA) is gaining attention as an ideal material for label-free colorimetric biosensor development due to its unique color-changing properties in response to external stimuli. PDA forms through the self-assembly of diacetylene monomers, with color change occurring as its conjugated backbone twists in response to stimuli such as temperature, pH, and chemical interactions. This color change enables the detection of biomarkers, metal ions, and toxic compounds. Moreover, the combination of PDA with polymeric structures including hydrogels further enhances the sensitivity and structural stability of PDA-based biosensors, making them reliable and effective in complex biological and environmental conditions. This review comprehensively examines recent research trends and applications of PDA–polymeric structure hybrid biosensors, while discussing future directions and potential advancements in this field.

## 1. Introduction

The growing demand for accurate and efficient detection technologies has positioned biosensors as indispensable tools for addressing challenges across a range of sectors. These biosensors combine biological recognition elements with physical transducers to detect specific substances and convert the results into measurable signals, with applications across various fields such as medical diagnostics, environmental monitoring, and food safety [1]. In particular, for field suitability, label-free, colorimetric biosensors, which are simple and cost- and time-effective, have gained attention [2,3]. Among these, polydiacetylene (PDA) has been consistently applied in biosensor development research due to its unique color-changing properties [4].

Polydiacetylene (PDA) is a polymer formed through the self-assembly and subsequent polymerization of diacetylene monomers, exhibiting a characteristic color-change response to external stimuli [5]. This color-change property results from the twisting of the unique conjugated structure of the PDA backbone, a process induced by various external factors such as temperature, pH, and interactions with specific chemicals [6,7]. Notably, the head group of diacetylene can be functionalized to serve as a bioreceptor, conferring specificity in detecting particular biochemical substances [8]. Initially, untwisted PDA appears blue, but upon exposure to specific stimuli, the twisting of the backbone leads to a color transition to red, which can be utilized as a detection signal. For these reasons, PDA is considered an ideal material for the development of label-free, colorimetric sensors and is actively studied as an innovative material for biosensors [9,10].

In biosensors, PDA serves both as a receptor for detecting biomarkers and as a physical transducer that converts this detection into a colorimetric signal [11]. The colorimetric signal change exhibited by PDA is sensitive and distinct, making it an effective tool for detecting a wide range of analytes, such as biomolecules [12], metal ions [13], and toxic compounds [14]. For instance, PDA-based biosensors are designed to undergo color changes upon binding with specific biomarkers, with the sensitivity of the color change varying proportionally with the analyte concentration. This enables PDA-based biosensors to function as rapid and reliable tools across diverse diagnostic fields [15,16]. Additionally, the high sensitivity and colorimetric detection capabilities of PDA-based sensors distinguish them from conventional sensors, making them more intuitive and user-friendly analytical tools.

Extensive research on biosensors employing PDA has been conducted across various fields. Previous researchers have published a range of review papers that summarize and analyze PDA-related research within specific fields, offering direction for subsequent studies. Existing reviews cover PDA’s unique structure and properties comprehensively [17,18], while others focus on enhancing PDA’s sensitivity [19] or functionalization [20]. Additionally, review articles have been published on application fields such as food [21], biomedical fields [22], and environmental monitoring [8], as well as on specific targets like volatile organic compounds (VOCs) [23], and microorganisms [24,25]. Furthermore, a dedicated review on mechanochromism, one of PDA’s inherent properties, has also been published [26].

On the other hand, PDA can be combined with 3D substrate structures, such as polymeric network structures, which have not been directly addressed in previous review articles. Polymeric structures provide a network architecture that stably disperses and retains PDA. Among these, prominent polymeric networks like hydrogels offer high water retention and excellent biocompatibility, playing a crucial role in enhancing the response rate and sensitivity of PDA-based biosensors while maintaining the structural stability of biomolecules [27,28]. Furthermore, incorporating PDA into polymeric network structures already used in various fields, such as medical devices [29] and food packaging [30], can enhance user-accessibility. These advantages have led to a number of studies integrating PDA with polymeric network structures, thus broadening the application scope of biosensors. However, a review specifically addressing this topic in detail has not yet been reported.

This review aims to comprehensively examine research trends in biosensors that combine PDA with polymeric network structures, summarizing how different polymers are applied across different application fields (Table 1). Finally, by exploring the future research directions and potential advancements of PDA-based biosensors, this review seeks to offer valuable insights for researchers in this field.

## 2. PDA with Natural Polymer-Based Network Structure

Natural polymeric structures, including agarose, alginate, and chitosan, offer significant advantages in terms of cost-efficiency, biocompatibility, and eco-friendliness when combined with polydiacetylene (PDA) for biosensor applications [56,57]. These polymers, derived from natural sources, are inexpensive, suitable for mass production, and ensure stable interactions with biological materials due to their excellent biocompatibility. Furthermore, their high biodegradability minimizes their environmental impact when used as environmental sensors. This chapter introduces several applications of natural polymeric structures in PDA-based biosensor development and describes the characteristics of each application example.

### 2.1. Agarose

**Figure 1:**
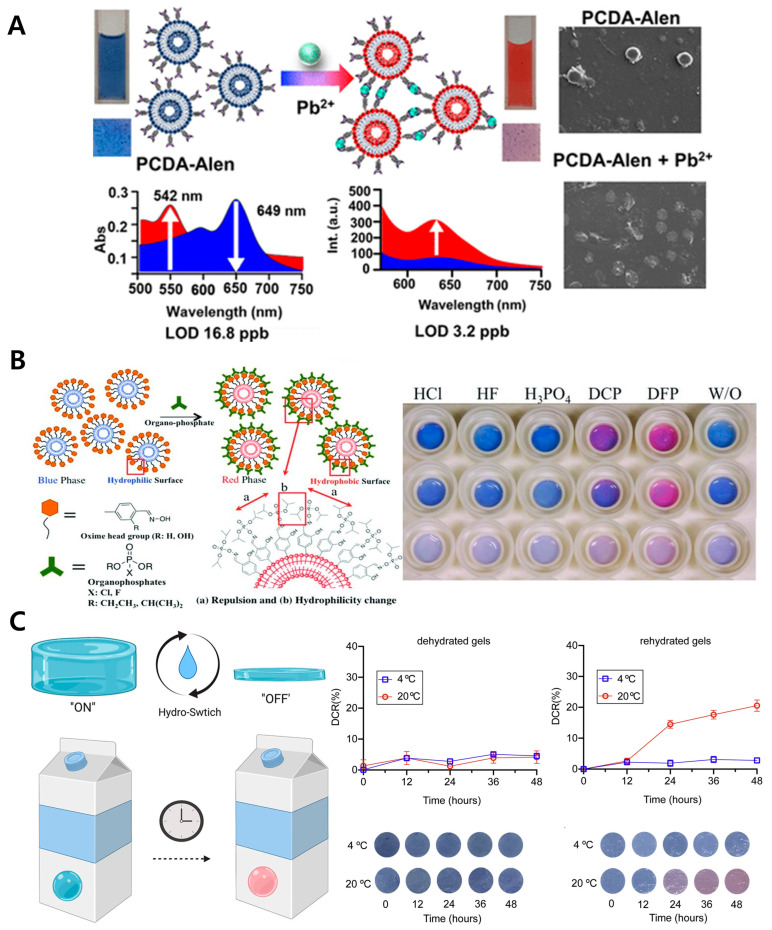
PDA-based biosensor with agarose polymer. (**A**) Schematic illustration showing the detection mechanism of Pb^2+^ using an alendronate–PDA–agarose matrix, alongside images of sensor color changes and quantitative analysis graphs (left). SEM images of the sensor surface before and after Pb^2+^ exposure (right). Reprinted with permission from [31]. Copyright 2024 American Chemical Society. (**B**) Schematic illustration of the detection mechanism for warfare gases using oxime-functionalized PDA sensors embedded in agarose gel (left) and images showing color changes in oxime–PDA-embedded agarose gel upon exposure to warfare gases (right). Reprinted with permission from [32]. Copyright 2012 John Wiley and Sons. (**C**) On–off switching mode of a PDA–agarose gel-based milk freshness indicator (left), along with images showing color changes under different storage conditions for each mode and corresponding quantitative color change graphs (right). Reprinted with permission from [33]. Copyright 2021 Elsevier.

Agarose, a naturally derived polysaccharide, possesses excellent transparency and higher pH stability than other natural polymers, making it suitable for the development of stable biosensors [58]. This material is known for its unique thermoreversible gelation property [59], where it dissolves at high temperatures and transitions to a gel state upon cooling at specific concentrations. And this polymer matrix provides ample hydrophilic space, facilitating substance diffusion. Due to these physicochemical properties, agarose combined with polydiacetylene (PDA) can be utilized in diverse environmental and biological sensing applications (Figure 1). For instance, a sensor was developed using alendronate as a receptor on a PDA sensor to detect mercury in tap and rainwater, where the interaction between mercury and alendronate induced a color change in PDA, enabling mercury concentration detection (Figure 1A) [31]. Additionally, a sensor using oxime as a receptor was developed to detect the nerve toxin DFP in the air, with DFP-oxime binding causing structural changes in PDA that resulted in a color shift (Figure 1B) [32]. In a case where no receptor functionalization was applied, the proliferation of bacteria in milk produced fatty acids that induced a color change in PDA, allowing for the detection of milk contamination (Figure 1C) [33,34]. In this study, the authors developed and applied an image-based digital color analysis software using the Python programming language. Similarly, another study used unfunctionalized receptors to detect bacterial growth in blood via color changes caused by secondary metabolites from bacterial proliferation [35]. These studies demonstrate that the combination of agarose and PDA can function effectively as biosensors for biomarker detection in diverse environments. While agarose has the advantage of not requiring additional chemicals for gelation, thereby minimally affecting PDA’s color change, it has the drawback of relatively low durability.

### 2.2. Alginate

**Figure 2:**
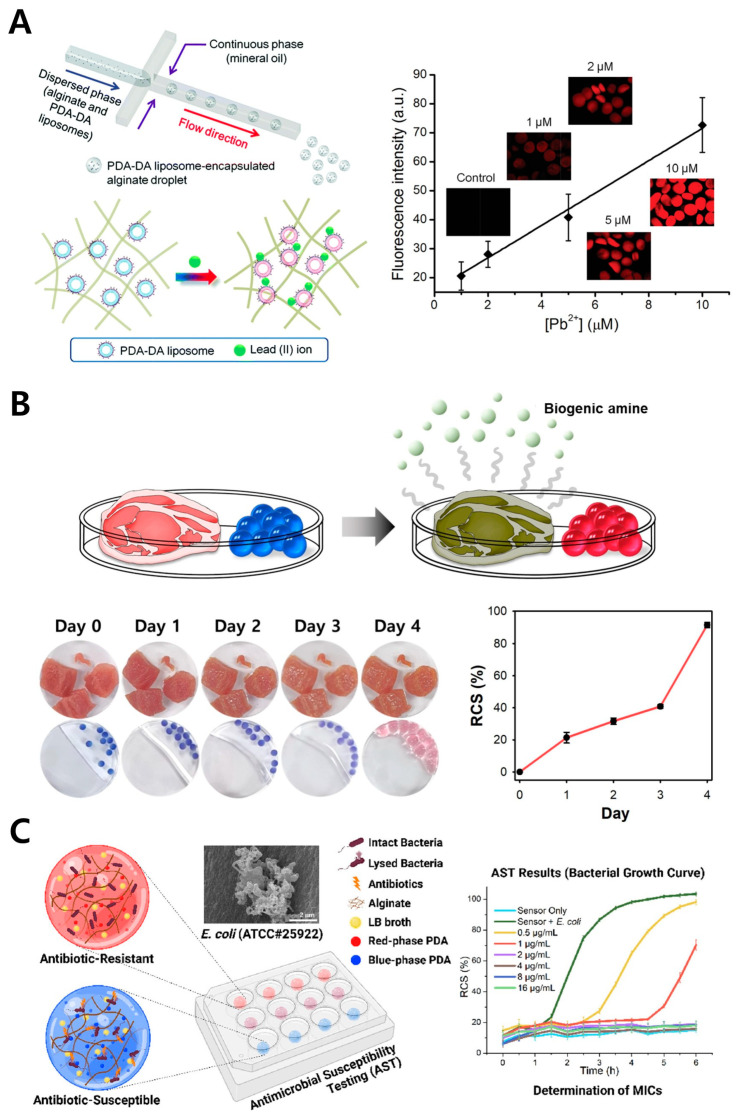
PDA-based biosensor with alginate polymer. (**A**) Schematic illustration of the fabrication of dopamine–PDA-encapsulated alginate beads and their detection of Pb^2+^ (left), along with images showing fluorescence intensity changes at different Pb^2+^ concentrations and quantitative analysis graphs (right). Reprinted with permission from [39]. Copyright 2015 Royal Society of Chemistry. (**B**) Schematic illustration of freshness monitoring through the detection of biogenic amines in meat using NHS functionalized PDA–alginate hydrogel beads (upper), and images showing color changes in the PDA–alginate biosensor and corresponding quantitative graphs as meat spoils (bottom). Reprinted with permission from [40]. Copyright 2023 Elsevier. (**C**) Schematic illustration of the color-change mechanism in the PDA–alginate biosensor caused by the growth of pathogenic bacteria and its application in antimicrobial susceptibility testing (AST) (left). Quantitative graph showing differences in color changes in the PDA–alginate biosensor based on antibiotic concentrations (right). Reprinted with permission from [41]. Copyright 2023 American Chemical Society.

Alginate is a hydrophilic polymer composed of polysaccharides and is naturally found as a component of the cell walls in brown algae [60,61]. In brown algae, it provides structural stability and serves as a protective barrier, allowing the organism to survive in harsh marine environments. Alginate has the unique ability to form cross-links with metal ions, resulting in gelation. This gelled alginate can immobilize multiple hydrophilic substances, allowing material exchange through the spaces within the polymer, which has led to its wide application in drug delivery systems [62,63], tissue engineering [64,65], and biosensors [66,67]. In the biosensor field, PDA nanoparticles, as hydrophilic nanomaterials, can be stably immobilized within alginate polymers, leading to several reported applications (Figure 2) [36,37,38]. When alginate polymers are exposed to aqueous solutions containing heavy metals, the metal ions diffuse into the polymer. A sensor can be designed with PDA fixed in the polymer network that selectively binds specific metal ions and induces a color change in response. Based on this, an alginate polymer sensor for monitoring the presence of lead in aqueous solutions was developed (Figure 2A) [39]. Gases in the air can also diffuse into alginate polymers. An alginate polymer was developed to capture PDA functionalized with a receptor that selectively recognizes biogenic amines, gases released during food spoilage, to monitor food freshness (Figure 2B) [40]. A sensor was also developed to detect pathogen growth and simultaneously screen antibiotics by immobilizing unfunctionalized PDA within the alginate polymer (Figure 2C) [41]. In this study, the PDA–alginate sensor could be stored in a dry state and, upon the addition of a liquid analyte, could be rehydrated and exhibit color-change functionality. This demonstrates its portability and potential for long-term storage. The color-changing ability of PDA immobilized within alginate can be influenced by the metal ions used for gelation. Utilizing this property, an alginate polymer was developed to exhibit irreversible color changes at specific temperatures, enabling application as a temperature-monitoring sensor [42]. Lastly, the relatively easy control of alginate gelation allows the use of microfluidic systems to create sensors of specific shapes and sizes. However, care should be taken as the metal ions required for gelation may excessively influence PDA’s color change.

### 2.3. Chitosan

**Figure 3:**
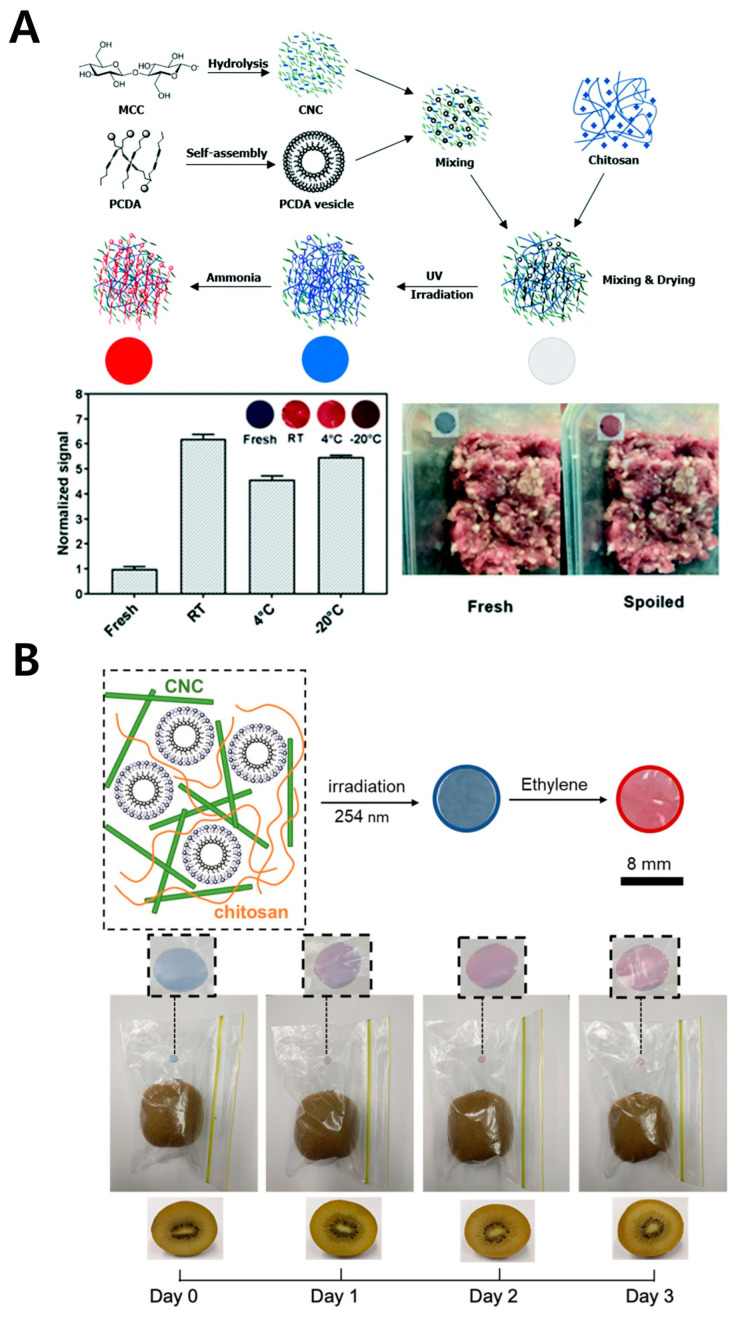
PDA-based biosensor with chitosan polymer. (**A**) Schematic illustration of the fabrication process of the chitosan-embedded PDA biosensor (upper). Images showing color changes in the chitosan–PDA biosensor when exposed to spoiled meat, along with corresponding quantitative analysis graphs (bottom). Reprinted with permission from [45]. Copyright 2019 Royal Society of Chemistry. (**B**) Schematic illustration of ethylene detection using a PDA-encapsulating chitosan biosensor for food freshness monitoring (upper). Images showing color changes in the PDA–chitosan biosensor in response to ethylene gas released during kiwi ripening (bottom). Reprinted with permission from [46]. Copyright 2020 American Chemical Society.

Chitosan, a versatile natural polymer with biocompatibility, biodegradability, and antimicrobial properties [68], is used in a wide range of applications, from food packaging to biosensors [69,70]. When introduced into PDA-based sensors, chitosan enhances the mechanical properties of the film and increases stability, enabling the development of flexible and durable sensors. One of the primary manufacturing methods for PDA–chitosan sensors involves drying a PDA–chitosan mixture solution to form a film. This process begins with preparing a PDA dispersion in an aqueous solvent, which is then added to a chitosan solution and mixed. The mixture undergoes ultrasonic treatment to remove bubbles, and the solvent is evaporated to create a film. Such flexible film sensors can be easily used for food monitoring by being compatible with packaging materials (Figure 3) [43,44]. For instance, an ammonia gas detection sensor can be developed using this method (Figure 3A) [45]. A film sensor combining PDA, chitosan, and cellulose nanocrystals (CNCs) was shown to operate over a wide temperature range from −20 °C to room temperature, proving useful for spoilage detection in low-temperature food storage. Additionally, a similar method was used to develop an ethylene gas detection sensor (Figure 3B) [46]. In this study, the carboxyl groups on PCDA were replaced with thiol groups and mixed with chitosan and CNC to produce a film sensor. This sensor not only effectively detected ethylene released during fruit ripening but also allowed easy visual observation of color change, making it suitable for applications in the food industry. Furthermore, the layer-by-layer (LbL) assembly method enables the fabrication of sensors with more refined structures [71]. This technique involves alternately layering materials with opposite charges onto a substrate. In LbL assembly with PDA and chitosan, negatively charged PDA vesicle solutions and positively charged chitosan solutions are alternately dipped to build up layers. The LbL method is beneficial because it allows control of color intensity by adjusting the number of layers. Using this method, a sensor for detecting water-soluble aromatic compounds was developed. This multilayer film, created by alternately layering PDA vesicles and chitosan, effectively detected compounds like benzoic acid and nitrophenol through interactions with α-cyclodextrin. Due to its excellent durability and natural antimicrobial properties, chitosan has been commonly used in food packaging for long-term storage. However, given that it is only stable within specific pH ranges, its potential to lose stability under certain conditions must also be considered.

## 3. PDA with Synthetic Polymer-Based Network Structure

PDA-based biosensors utilizing synthetic polymers offer the advantage of easier control over physical and chemical properties compared to natural polymers [72]. Notable synthetic polymers applied with PDA include PVA (polyvinyl alcohol), PEG-DA (polyethylene glycol diacrylate), and PDMS (polydimethylsiloxane), each presenting several application potentials according to their unique characteristics. Synthetic polymers allow for precise design of pore size and mesh structure, enabling the tuning of sensor sensitivity and selectivity, which contributes to providing consistent performance across diverse environments [73]. This chapter lists different applications of synthetic polymeric structures in PDA-based biosensor development and summarizes the characteristics of each example.

### 3.1. Poly(Vinyl Alcohol) (PVA)

**Figure 4:**
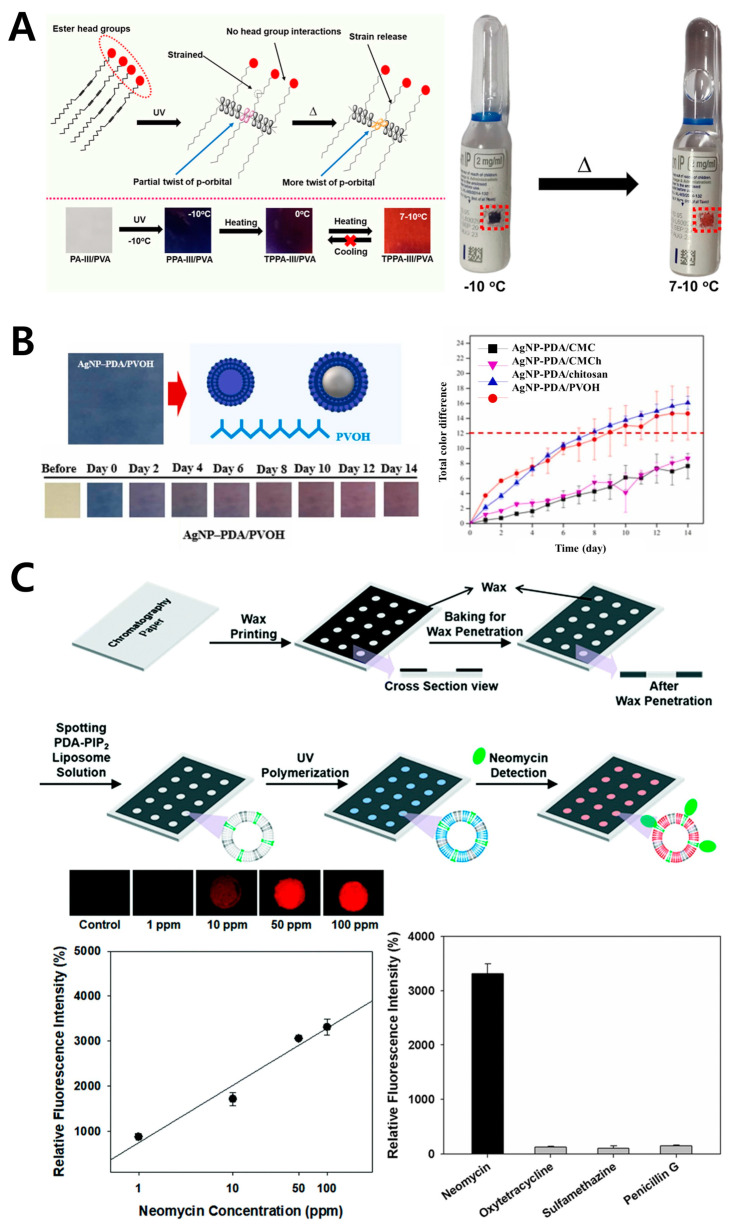
PDA-based biosensor with chitosan PVA polymer. (**A**) Schematic illustration showing the low-temperature indication mechanism of PDA (left upper) and images demonstrating the thermochromic properties of the PDA–PVA composite film (left bottom). Images of the PDA–PVA biosensor applied to a drug (vaccine) vial, showing a red color change when exposed to temperatures exceeding the threshold (right). Reprinted with permission from [48]. Copyright 2024 American Chemical Society. (**B**) Schematic illustration of the structure of the PDA–PVA biosensor for time–temperature indication (left upper). Images showing the sensor’s color change over time when exposed to temperatures outside the threshold (left bottom), along with a quantitative analysis graph of the color change over time (right). Reprinted with permission from [79]. Copyright 2024 Elsevier. (**C**) Schematic illustration of a neomycin probe functionalized PDA–PVA sensor film for neomycin detection (upper). Changes in the fluorescence intensity of the sensor based on the neomycin concentration, quantitative analysis graphs, and specificity verification results (bottom). Reprinted with permission from [49]. Copyright 2018 Royal Society of Chemistry.

PVA (poly(vinyl alcohol)) is a synthetic polymer well-known for its versatility and broad range of applications, widely used in fields such as biomedicine [74,75], agriculture [76], and biosensors [77]. The structure of PVA consists of repeating vinyl alcohol units capable of forming hydrogen bonds, resulting in excellent film-forming ability. Particularly, PVA’s hydrophilic properties allow it to readily incorporate multiple water-soluble materials like PDA. Furthermore, due to its flexibility and thermal and chemical stability, PVA is ideal for stably incorporating and protecting functional materials. PVA films offer a wide range of applications as sensors because of the ease with which their thickness, transparency, and durability can be controlled (Figure 4). The method of integrating PDA into a PVA matrix is similar to the approach with chitosan, where a PDA-containing aqueous solution is mixed with a PVA solution and then dried to form a film. Using this method, a PDA–PVA hybrid film was created and applied for ammonia gas detection [47]. Another application of PDA–PVA composite films has been reported as an irreversible low-temperature indicator [78]. Researchers incorporated PDA functionalized with different aliphatic alcohols into a PVA matrix to create a sensor that shows a color change at low temperatures above 7 °C (Figure 4A) [48]. This system can be applied to monitor temperature changes in frozen foods or pharmaceuticals. PVA films containing PDA supramolecular assemblies have also been developed as strip-type chemical sensors (Figure 4B) [79]. These researchers reported a color change in PDA triggered by specific molecular recognition, such as with α-cyclodextrin. PVA matrices are also known to enhance the stability of PDA sensors, aiding in the detection capability of target substances. In related studies, the importance of PVA was demonstrated when PDA liposomes were integrated into paper-based microfluidic devices. The addition of PVA significantly improved the stability of PDA liposomes on the paper substrate, contributing to increased sensor sensitivity for neomycin detection (Figure 4C) [49]. These studies highlight the critical role of PVA as a matrix material in PDA-based sensor systems. PVA’s flexibility and excellent durability enable the fabrication of a range of forms of sensors with PDA, providing characteristics suitable for practical applications. However, PVA’s sensitivity to humidity, which may weaken its mechanical properties upon moisture absorption, must be carefully considered in sensor development.

### 3.2. Poly(Ethylene Glycol) Diacrylate (PEG-DA)

**Figure 5:**
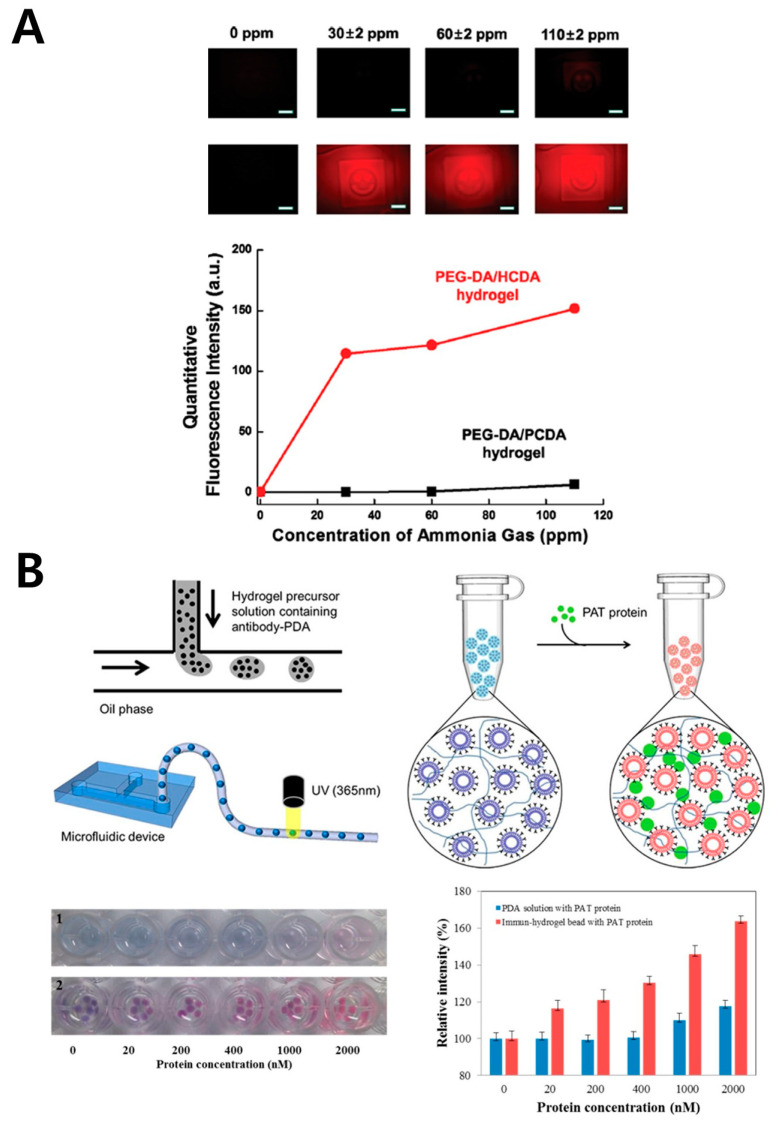
PDA-based biosensor with PEG-DA polymer. (**A**) PEG-DA–PDA biosensors for ammonia gas analysis. Images showing changes in fluorescence intensity with varying concentrations of ammonia (upper) and corresponding quantitative analysis graphs (bottom). Reprinted with permission from [50]. Copyright 2017 Springer Nature. (**B**) Fabrication process of a PEG-DA–Antibody–PDA hydrogel bead biosensor for GMO marker detection (upper left) and schematic illustration of PAT protein detection, a GMO marker protein (upper right). Images showing color changes in the hydrogel bead biosensor with varying PAT protein concentrations (bottom left) and corresponding quantitative analysis graphs (bottom right). Reprinted with permission from [51]. Copyright 2015 American Chemical Society.

PEG-DA (poly(ethylene glycol) diacrylate) is a polymer formed by combining polyethylene glycol (PEG) with diacrylate, creating a polymer network through photopolymerization upon exposure to UV light (365 nm) with a photoinitiator. The polymerized PEG-DA forms a porous network structure with inherent hydrophilic properties, allowing it to effectively trap hydrophilic substances [80]. Due to these characteristics, PEG-DA-based polymers are widely used in biomedical [81], tissue engineering [82], and biosensor [83] applications. PDA can also be synthesized as hydrophilic nanoparticles and encapsulated within PEG-DA polymers, allowing for biosensor applications (Figure 5). This encapsulation is achieved by mixing a PDA dispersion in an aqueous solution with PEG-DA, followed by 365 nm UV exposure, which traps PDA within the polymer structure. The resulting polymer structure permits the movement of several hydrophilic substances, chemicals, proteins, and gas molecules, demonstrating its potential as a biosensor [84]. A study has reported the development and optimization of PDA-encapsulated polymer structures exhibiting color changes in response to alpha-cyclodextrin, ammonia gas, and pH changes (Figure 5A) [50]. This structure has applications as a sensor for toxic gases, chemical detection, and food freshness monitoring. Another example is a PDA-encapsulated PEG-DA polymer sensor designed to detect specific proteins (Figure 5B) [51]. In this structure, PDA encapsulated within the polymer matrix was functionalized with antibodies to detect the GMO marker protein PAT, leading to the development of a detector for identifying GMO status in crops. Additionally, the integration of microfluidic systems enabled the creation of bead-shaped sensors, showcasing PEG-DA’s versatility in developing sensors of different forms. PEG-DA allows for the adjustment of structural properties by using PEG of different molecular weights and provides control over polymerization timing, facilitating the development of sensors tailored to specific purposes. However, the photoinitiators required for polymerization are toxic and may affect PDA’s color-changing properties, necessitating careful consideration in sensor development.

### 3.3. PDMS (Polydimethylsiloxane)

**Figure 6:**
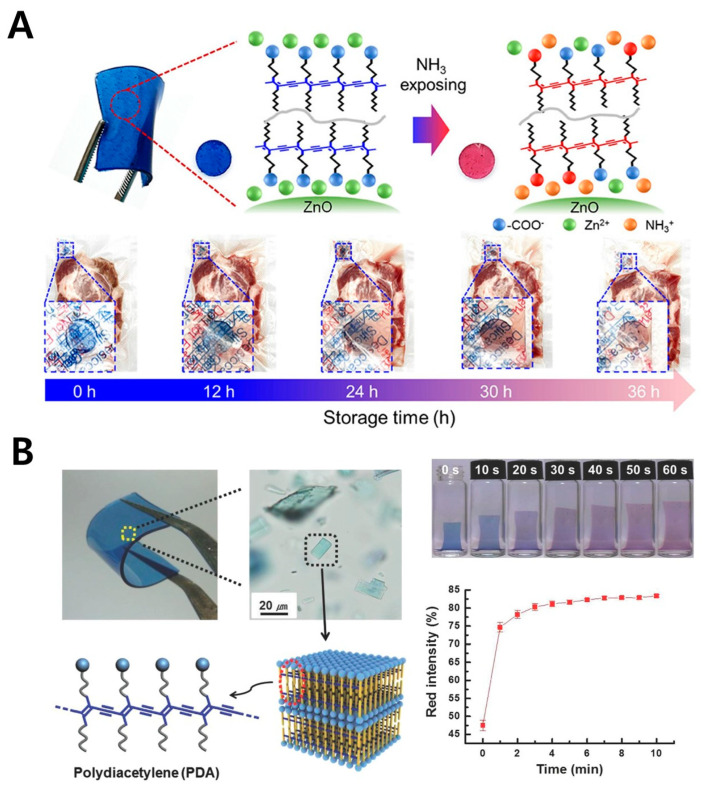
PDA-based biosensor with PDMS polymer. (**A**) Optical image of a PDA–ZnO-embedded PDMS film (upper right) and a schematic illustration of ammonia gas detection by the sensor (upper right). PDA–PDMS sensor images showing color change induced by ammonia gas emitted from decaying pork meat (bottom). Reprinted with permission from [52]. Copyright 2023 American Chemical Society. (**B**) Optical and microscopic images of a PDA–PDMS composite film sensor for hydrocarbon detection (upper left). Schematic illustration of PDA supramolecules encapsulated within the PDMS matrix (bottom left). Images showing the color change and swelling behavior of the PDA–PDMS sensor upon exposure to hydrocarbons over time (upper right) and a quantified graph of the color changes (bottom right). Reprinted with permission from [53]. Copyright 2014 John Wiley and Sons.

Due to its flexibility, transparency, chemical resistance, and ease of processing [85], PDMS (polydimethylsiloxane) is widely used across various fields, such as microfluidics [86], medical devices [87], electronics [88], and soft robotics [89]. Its biocompatibility and physical stability make it suitable for medical devices and implants, and its ability to form fine structures makes it a valuable material for lab-on-a-chip devices [90,91]. When combined with PDA, PDMS enables real-time monitoring of biological signals via color-change detection, making it applicable in diverse biosensors for disease diagnosis and food safety monitoring (Figure 6). One example is a color-changing ammonia sensor developed by incorporating polydiacetylene (PDA) and zinc oxide (ZnO) nanopellets into PDMS to detect meat spoilage (Figure 6A) [52]. This sensor exhibited a noticeable color change from blue to red even when exposed to concentrations of ammonia gas as low as 200 ppm. Furthermore, its thermal stability was demonstrated, as its color-changing characteristics were retained under exposure to elevated temperatures of up to 100 °C. Notably, these properties remained consistent for a duration of up to 180 days. In this study, the reaction kinetics were also thoroughly analyzed. Upon exposure to ammonia gas, the color changed rapidly within the first 2 h, with approximately 51.5% of the change occurring by 4 h. Considering its thermal stability and reaction kinetics, this sensor demonstrates significant potential for application in smart packaging systems to monitor the freshness of meat. PDMS–PDA colorimetric sensors have also been used as solvatochromic sensors that respond specifically to chloroform (Figure 6B) [53]. Upon exposure to chloroform, the sensor shifts from red to yellow due to weak hydrogen bonding with PDA, selectively detecting chloroform among 19 tested solvents. Another important application of PDMS is in detecting hydrocarbons through mechanochromic responses [92]. A PDMS film containing PDA expands upon exposure to saturated aliphatic hydrocarbons (SAHC), inducing mechanical stress on PDA and triggering a color change from blue to red. The sensor’s response varies based on the hydrocarbon chain length, enabling differentiation between compounds like pentane and heptane. This sensor system holds potential for practical applications, such as detecting fuel adulteration. PDMS’s gas permeability and hydrophobicity make it advantageous for developing sensors that detect gases or hydrophobic substances. Additionally, its durability makes it suitable for applications requiring long-term monitoring. However, it is less effective for detecting water-soluble substances, posing a limitation for certain applications.

## 4. Other Polymeric Structure: Diacetylene (DA) Polymerized Gel

Up to this point, only cases where PDA is encapsulated within an independent polymeric network structure have been discussed. In this chapter, two cases are addressed in which PDA forms a polymeric network structure on its own. The first case involves a PDA-incorporated peptide hydrogel, where DA is synthesized with specific amino acids that provide binding forces within the protein, resulting in a porous, reticular structure. The second case involves a PDA gel, where the porous gel structure is constructed primarily through light-induced crosslinking between DA monomers in PDA. While these types of DA polymerized gel-based biosensor offer the advantage of customizable design, their practical applications remain limited compared to other polymeric structures, as they have yet to be widely commercialized.

### 4.1. PDA-Incorporated Peptide Hydrogel

**Figure 7:**
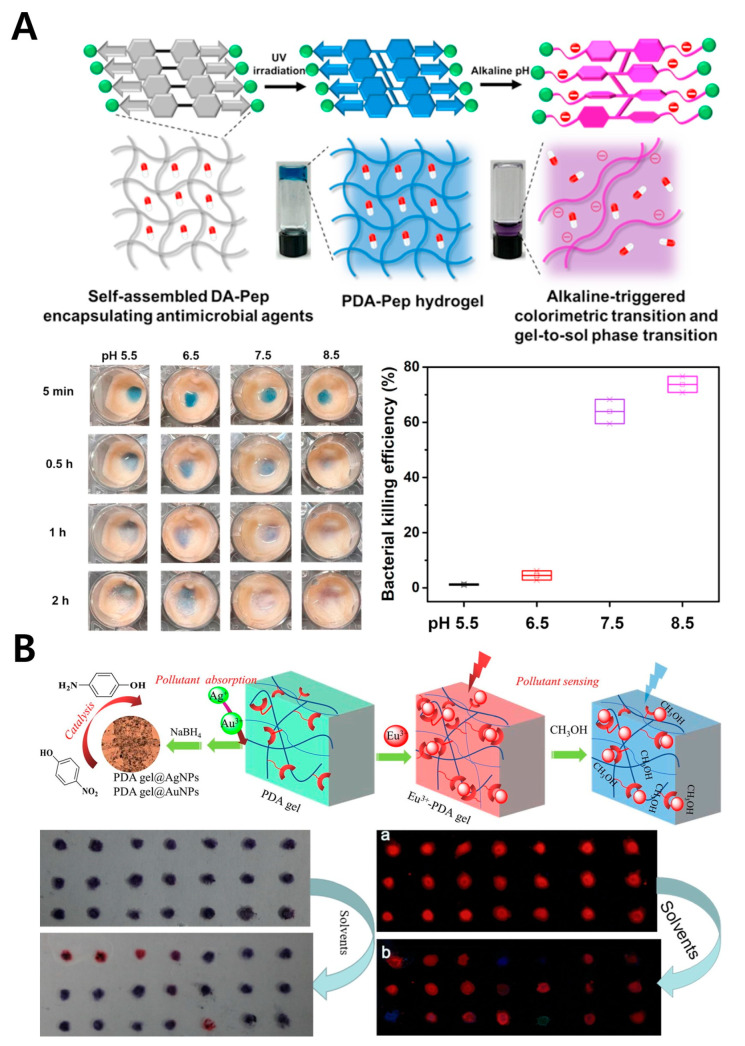
PDA biosensors with alternative polymeric structures. (**A**) Schematic illustration of a PDA–Pep hydrogel for pH sensing and on-demand antimicrobial release (upper). pH-responsive colorimetric transition of the PDA–Pep hydrogel applied to pig skin (bottom left). Graph showing the bacterial killing efficiency induced by the release of antibiotics from the PDA–Pep hydrogel (bottom right). Reprinted with permission from [54]. Copyright 2022 Elsevier. (**B**) Schematic illustration of the colorimetric response of the PDA gel to organic solvents (upper). Images showing the color changes and fluorescence intensity variations in the PDA gel upon exposure to various solvents (bottom). Reprinted with permission from [55]. Copyright 2021 Elsevier.

Peptides are biomolecules composed of short amino acid sequences, and it has been reported that specific amino acid sequences can be designed to form polymeric structures through self-assembly under certain conditions [93,94]. Researchers have applied this characteristic to PDA, proposing a novel PDA–peptide polymer (Figure 7A) [54]. They designed DA-pep, a peptide with a specific amino acid sequence capable of forming a polymer structure, linked to diacetylene (DA). When gelation occurs with DA-pep, antibiotics are incorporated within the structure, forming what is known as PDA–pep hydrogel. This study demonstrated that PDA-pep hydrogel not only detects alkaline pathogenic infections through color change, but also that as pH increases, the hydrogel breaks down as a result of repulsive interactions between individual fibers, resulting in a gel-to-sol transition, during which the antimicrobial peptides encapsulated in the hydrogel are slowly released and exert their antimicrobial activity. This finding confirms the versatile design possibilities of PDA and its potential to combine with peptide hydrogels rich in functional capabilities. While peptides offer the advantage of high-level molecular control, they also have limitations such as lower durability and higher costs.

### 4.2. PDA-Gel

PDA itself can form a network structure independently. A biosensor using a uniquely modified diacetylene to create a polymeric gel structure has been proposed (Figure 7B) [55]. This network structure, named PDA-gel, was formed solely from PDA without the addition of extra chemicals or catalysts for gelation. PDA-gel was shown to exhibit specific color changes in response to certain solvents, demonstrating the possibility of forming a gel structure using PDA alone and its potential applications in biosensing. However, similar to PDA-incorporated peptide hydrogel, PDA-gel has limitations such as low durability and high production costs, which necessitate careful consideration in sensor development.

## 5. Conclusions and Future Perspectives

Polydiacetylene (PDA) is gaining attention as an ideal material for the development of label-free colorimetric biosensors due to its unique property of changing color in response to external stimuli. PDA’s application in biosensors can be expanded through its combination with polymeric network structures as 3D substrate structures, which enhance its stability and promote PDA dispersion. In particular, the high water retention and biocompatibility of hydrogels, which are prominent polymeric structures, ensures the structural stability of internal biomolecules, ultimately improving the response rate and sensitivity of PDA-based biosensors.

This review provides a comprehensive analysis of the potential applications, advantages, and disadvantages of biosensors utilizing PDA in combination with a range of natural and synthetic polymer structures. Natural polymers like agarose, alginate, and chitosan offer cost-efficiency, excellent biocompatibility, and environmental friendliness due to their biodegradability. These polymers are cell-friendly and interact stably with biological materials, making them advantageous for environmental and biological sensors. However, they present limitations in long-term sensor reliability due to challenges in controlling physical properties and lower durability. In contrast, synthetic polymers such as PVA, PEG-DA, and PDMS enable control over sensitivity and selectivity through superior mechanical strength and material control, providing stable sensor performance in several forms and environments. Although synthetic polymers are less biodegradable compared to natural polymers, they offer the advantage of optimizing interactions with different substances through highly controlled properties. Peptide and PDA self-assembled structures offer potential for customized sensor development through precise molecular design, but they face constraints in terms of cost and durability.

In future research, the development of composite polymers to enhance biocompatibility, environmental friendliness, and performance consistency will emerge as a crucial direction. For instance, hybrid structures combining the biocompatibility of natural polymers with the physical durability of synthetic polymers could provide both eco-friendliness and high durability. Additionally, combining these structures with PDA is expected to improve the sensitivity of colorimetric changes in sensors, while advanced functionalization techniques could lead to the development of highly reliable sensors capable of more effectively detecting specific biomarkers. The integration of digital image analysis and real-time data processing through artificial intelligence is also anticipated to maximize the potential of PDA-based biosensors.

## Figures and Tables

**Table 1:** Comparison of polymeric structures in PDA-based biosensors.

Polymer Type	Specific Polymer	Target Type	Specific Target	Limit of Detection	Practical Application	Application Area	Ref.
Natural polymer	Agarose	Heavy metal	Pb^2+^	3.2 ng/mL	Environmental water source	Environment	[31]
		Warfare gas	DFP	160 mg/m^3^	N/A	Safety	[32]
		Spoilage marker	FFA	N/A	Milk	Food	[33,34]
		Contamination marker	Pathogenic bacteria	10^2^ cells	Blood, urine	Healthcare	[35]
	Alginate	Disease marker	BPAs	1.4 ng/mL	Serum, urine	Healthcare	[36]
		Organic solvent	IPA	32 mg/mL	N/A	Environment	[37]
		Heavy metal	Pb^2+^	200 ng/mL	N/A	Environment	[38,39]
		Spoilage marker	BAs	100 ng/mL	Pork meat	Food	[40]
		Infection marker	Pathogenic bacteria	10 CFU/mL	Blood	Healthcare	[41]
		Freshness indicator	Temperature	N/A	N/A	Food	[42]
	Chitosan	Infection marker	pH	N/A	N/A	Healthcare	[43]
		Waste marker	Aromatic compounds	20 mM	N/A	Environment	[44]
		Spoilage marker	Ammonia gas	300 μg/mL	Beef product	Food	[45]
		Food quality indicator	Ethylene	600 μg/mL	Kiwi	Food	[46]
Synthetic polymer	PVA	Spoilage marker	Ammonia gas	1000 μg/mL	Beef hairtail	Food	[47]
		Storage stability indicator	Temperature	N/A	Vaccine vial	Healthcare	[48]
		Antibiotics	Neomycin	1 μg/mL	N/A	Healthcare	[49]
	PEG-DA	Spoilage marker	Ammonia gas	N/A	N/A	Environment	[50]
		GMO marker	PAT protein	20 nM	N/A	Food	[51]
	PDMS	Spoilage marker	Ammonia gas	160 μg/mL	Pork, chicken	Food	[52]
		Hydrocarbon	Kerosene	N/A	Diesel oil	Industrial	[53]
Others	Peptide gel	Infection marker	pH	10 μM	Pig skin	Healthcare	[54]
	PDA gel	Waste marker	Organic solvent	12.5 mM	N/A	Environment	[55]

## Data Availability

No new data were created or analyzed in this study.
